# Supplementary figures and images for: Global chromatin mobility induced by a DSB is dictated by chromosomal conformation and defines the HR outcome
Source: eLife. 2022 Sep 20;11:e78015. doi: 10.7554/eLife.78015 (PMC9489209; doi:10.7554/eLife.78015)

# Fig. 1E

60h

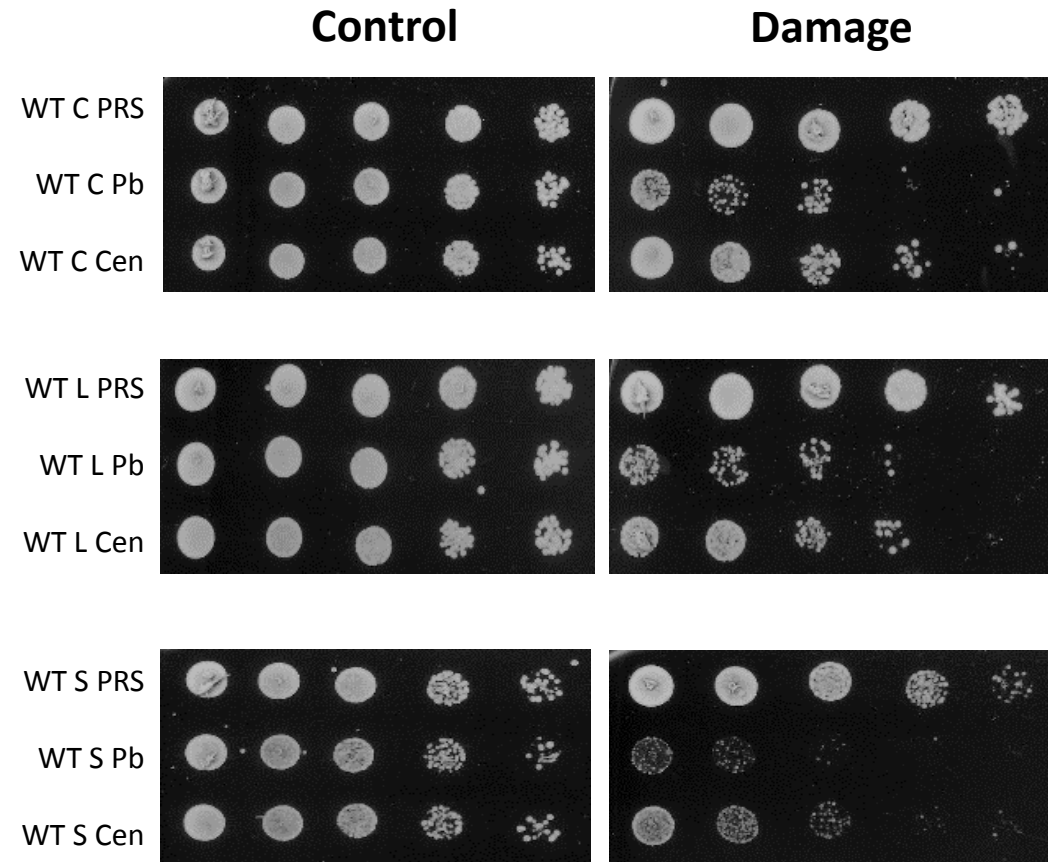

Supplement: Figure 1—source data 1. [file elife-78015-fig1-data1.pdf]

## Slide 1
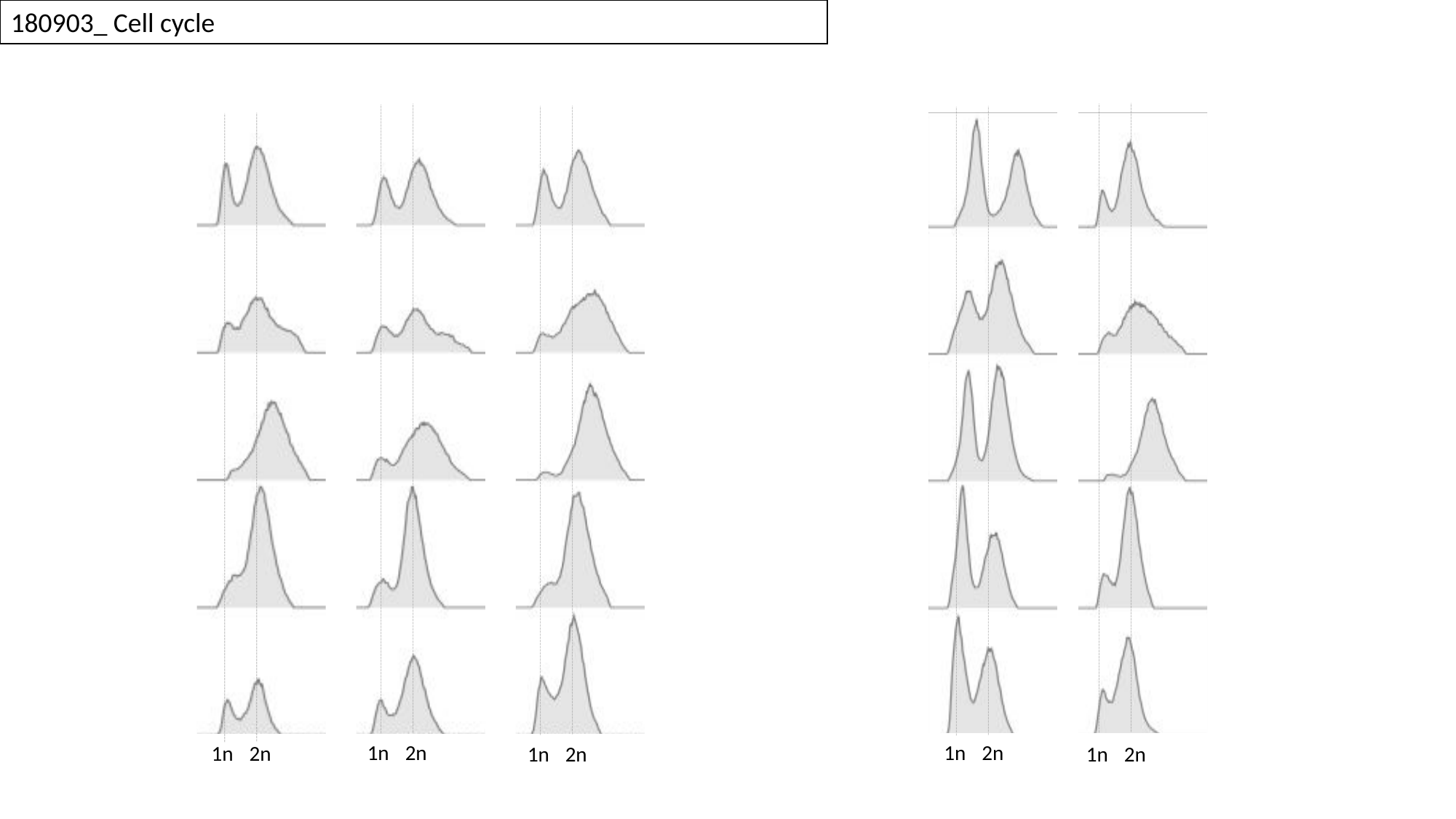

180903_ Cell cycle
1n
2n
1n
2n
1n
2n
1n
2n
1n
2n

Supplement: Figure 1—source data 2. [file elife-78015-fig1-data2.zip › Source FACS1 FiGURES/Figure 1D-source data 3_180903/20180903_Cell cycle_YEF1202_YEF1203_YEF1205_figures .pptx]

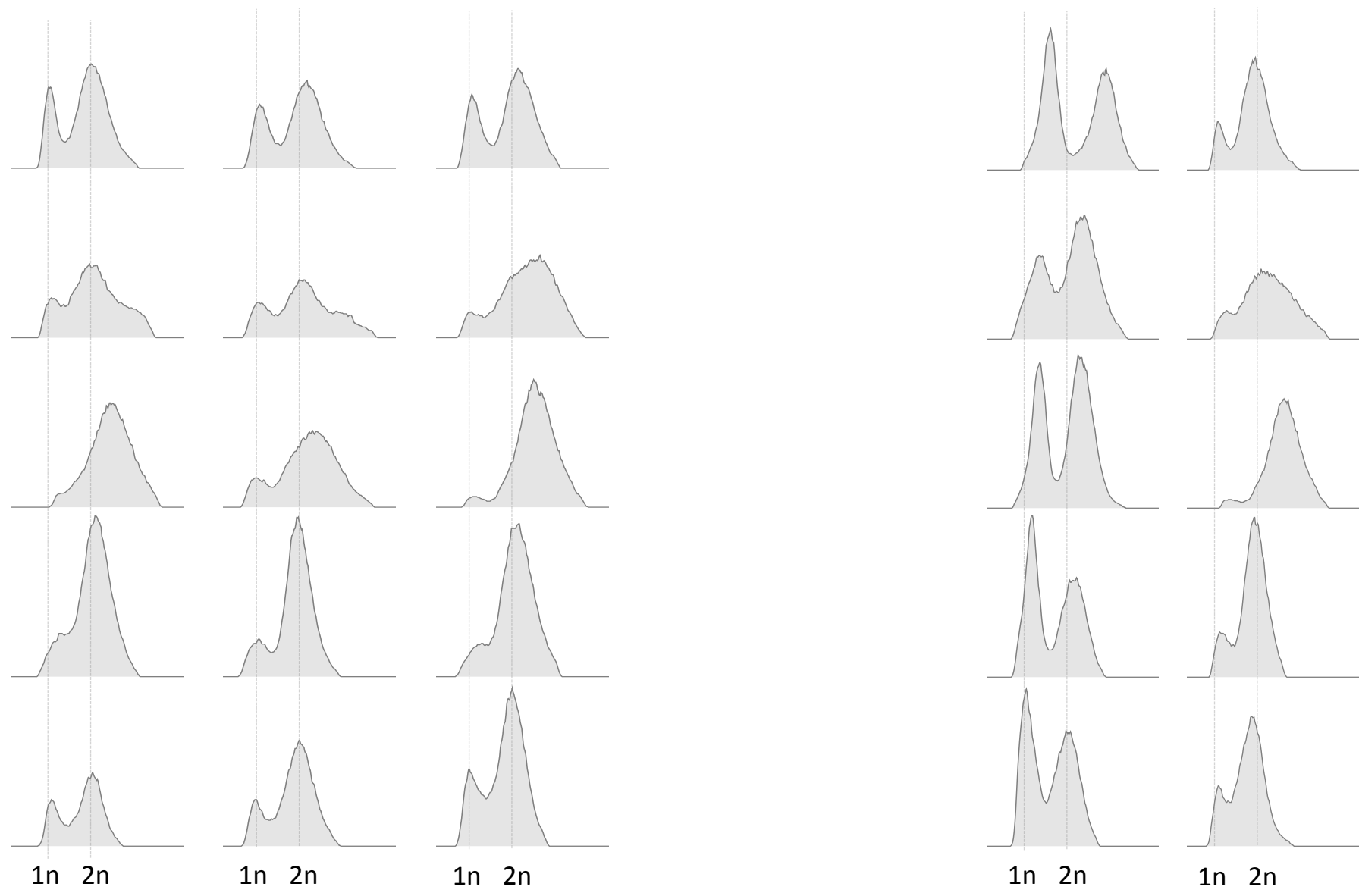

Supplement: Figure 1—source data 2. [file elife-78015-fig1-data2.zip › Source FACS1 FiGURES/Figure 1D-source data 3_180903/Figure 1D-source data 4.pdf]

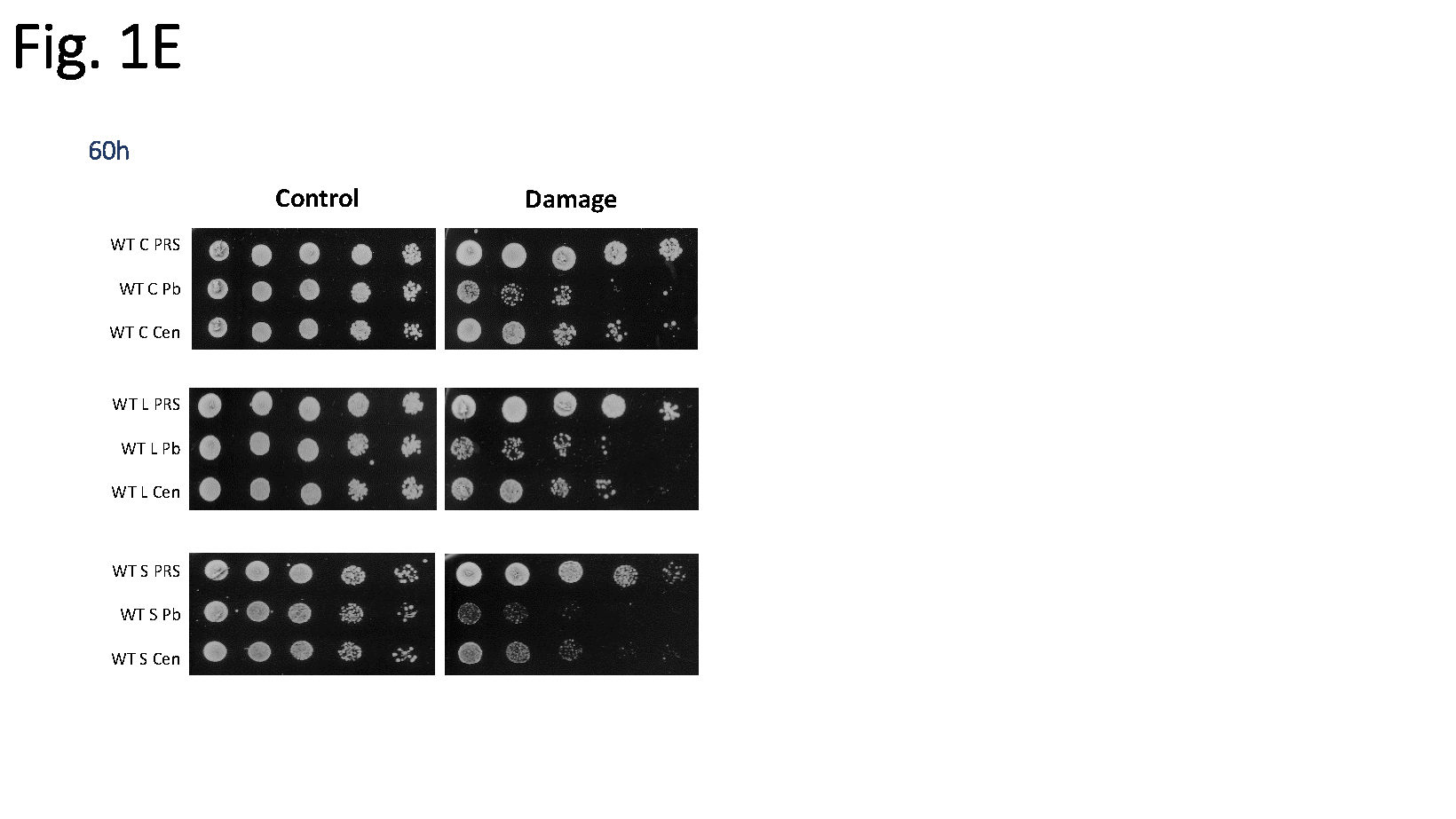

Supplement: Figure 1—source data 3. [file elife-78015-fig1-data3.zip › F1Sd3.jpg]

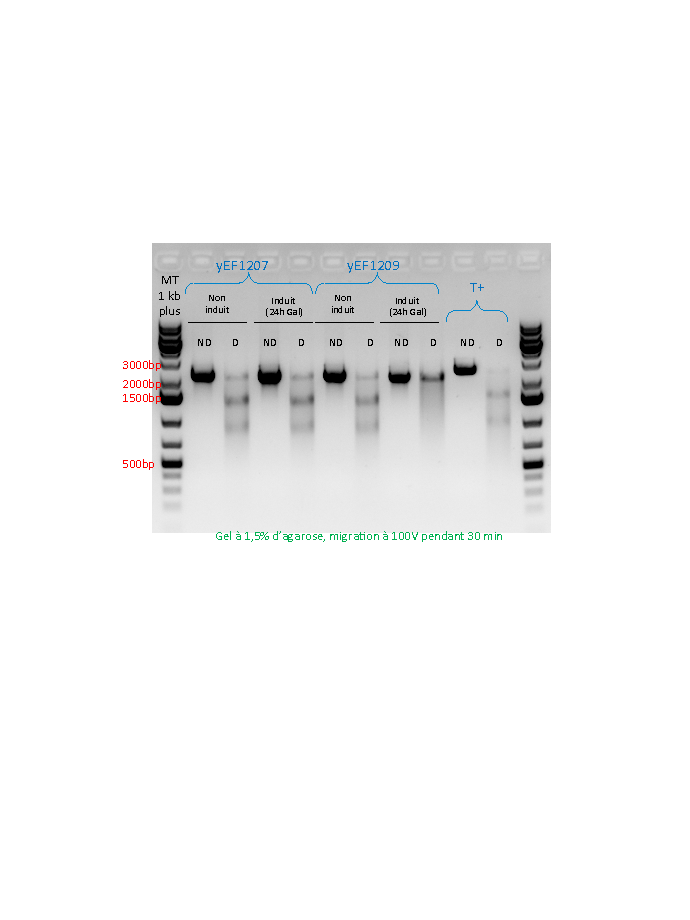

Supplement: Figure 1—figure supplement 1—source data 4. [file elife-78015-fig1-figsupp1-data4.zip › S1.jpg]

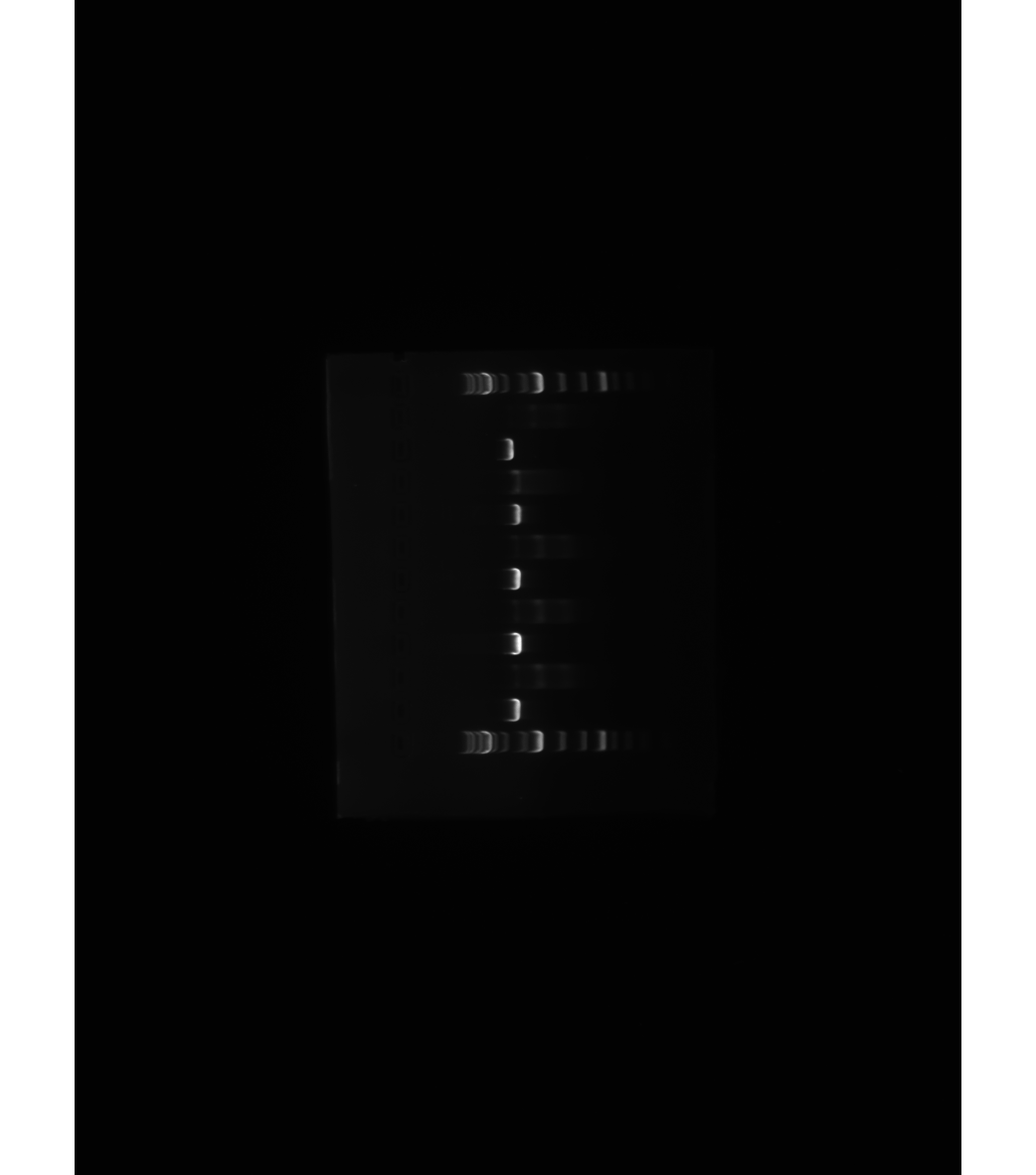

Supplement: Figure 1—figure supplement 1—source data 4. [file elife-78015-fig1-figsupp1-data4.zip › S2.jpg]

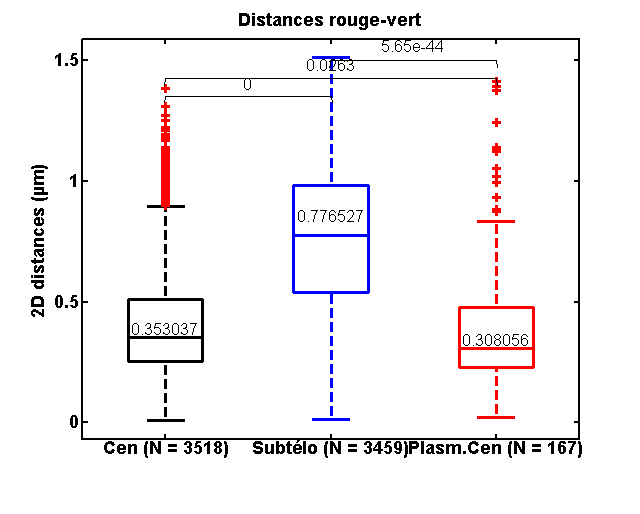

Supplement: Figure 1—figure supplement 1—source data 5. [file elife-78015-fig1-figsupp1-data5.zip › 8d8ee659-3844-4116-8c84-091c07076958.tif]

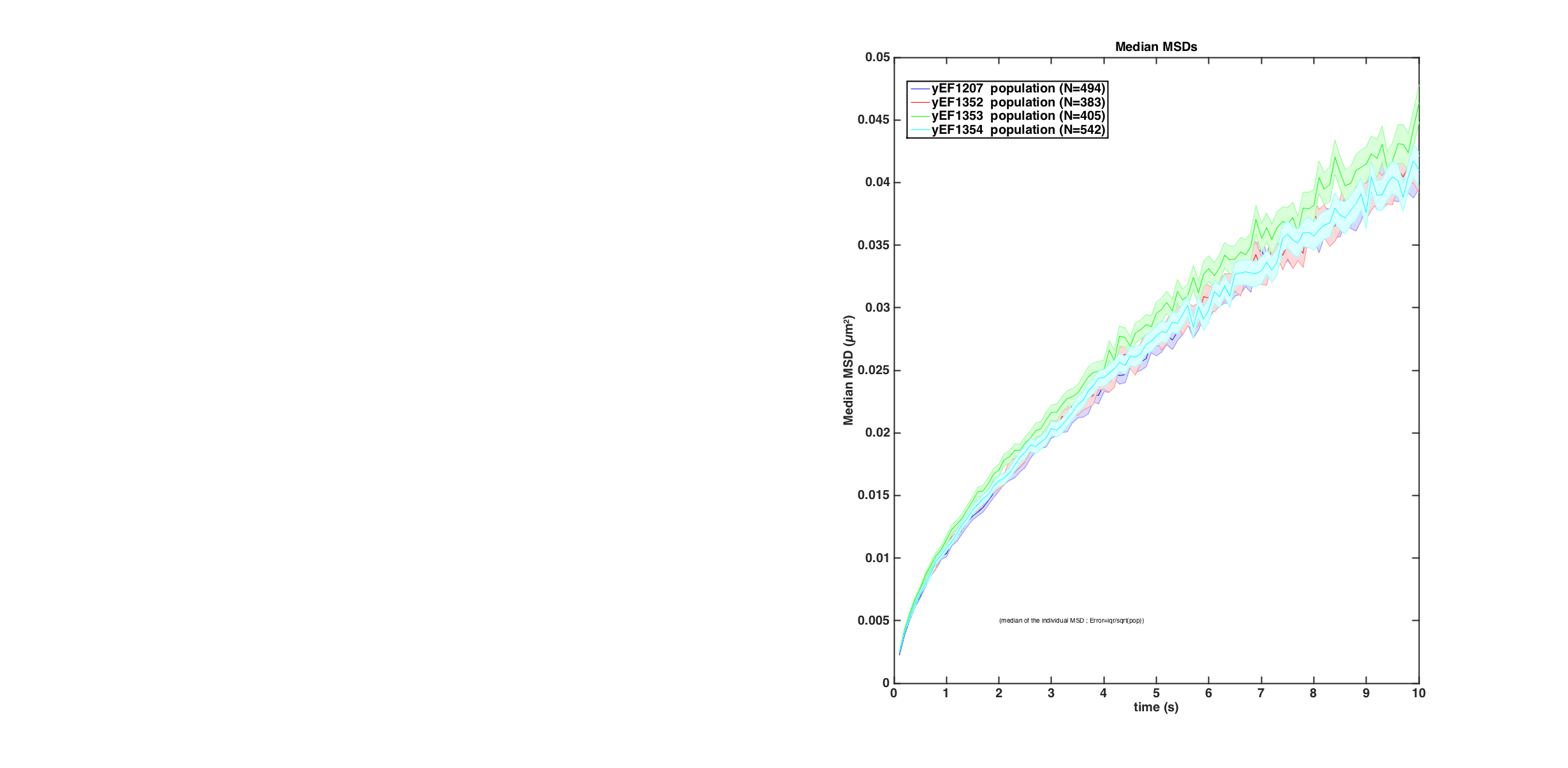

Supplement: Figure 2—source data 1. [file elife-78015-fig2-data1.zip › 1.jpg]

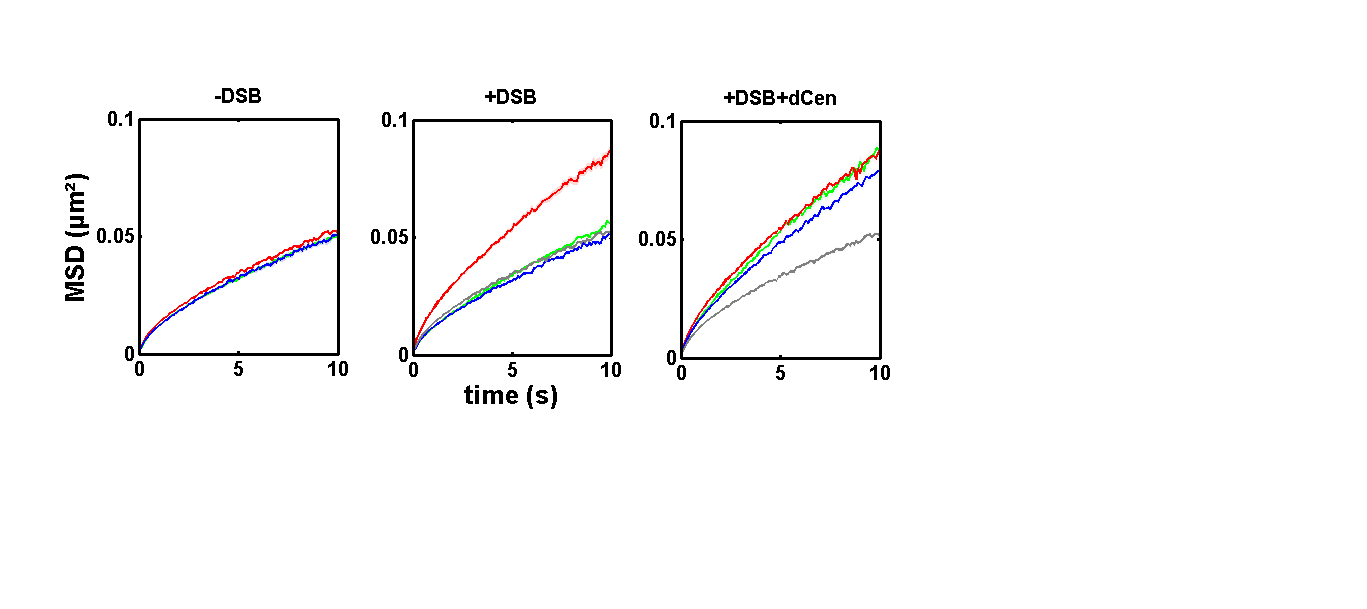

Supplement: Figure 2—source data 1. [file elife-78015-fig2-data1.zip › 2.jpg]

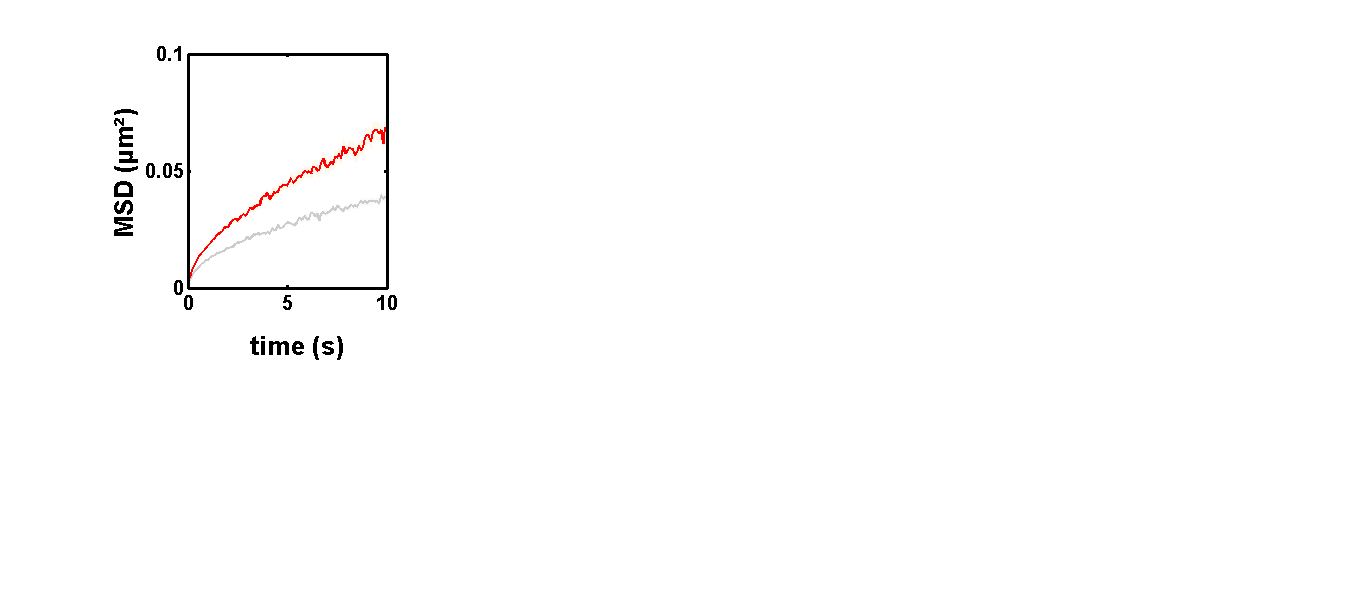

Supplement: Figure 2—source data 2. [file elife-78015-fig2-data2.zip › 1.jpg]

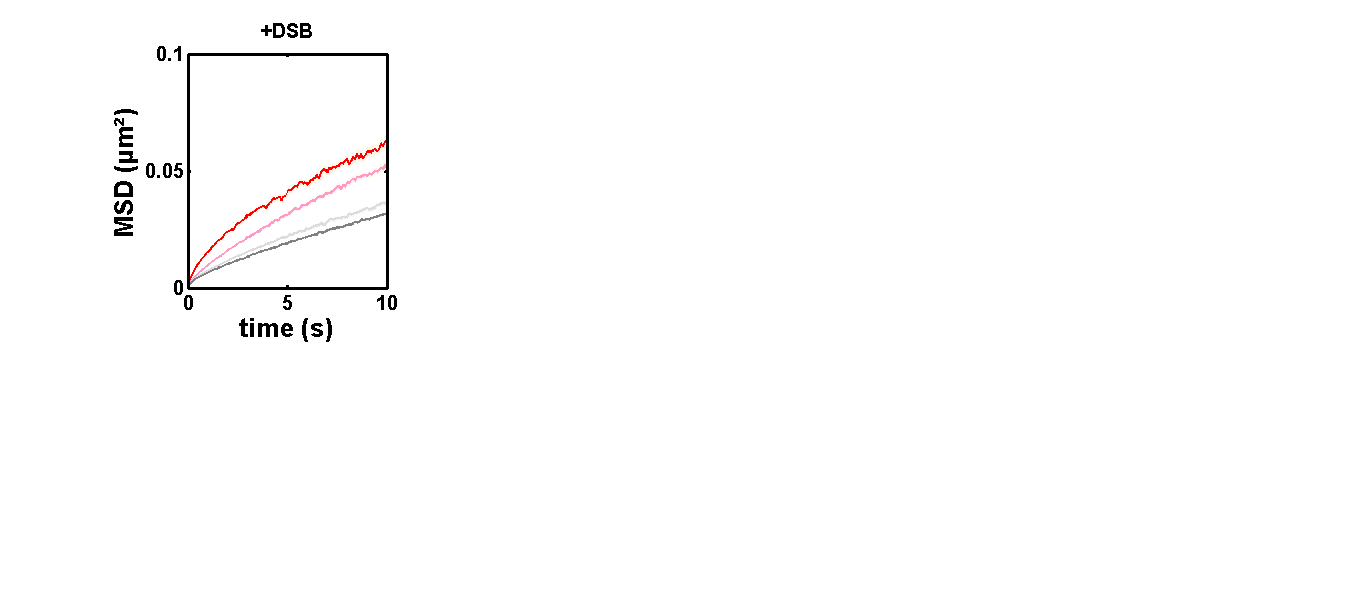

Supplement: Figure 2—source data 2. [file elife-78015-fig2-data2.zip › 2.jpg]

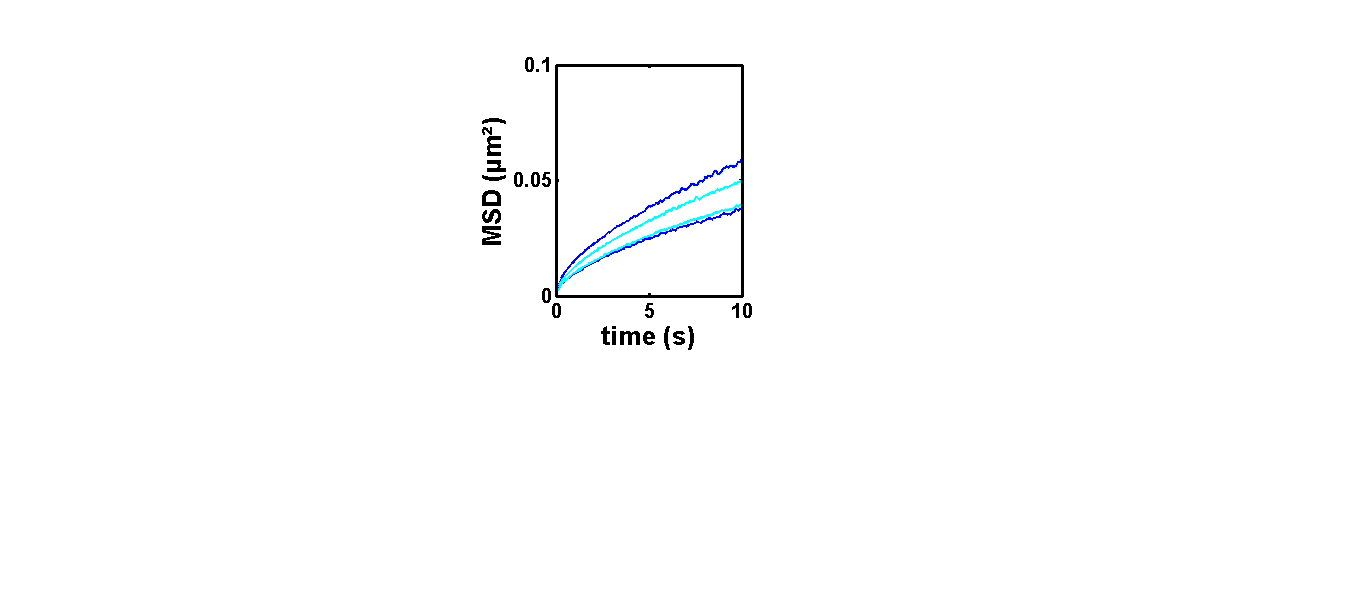

Supplement: Figure 2—source data 2. [file elife-78015-fig2-data2.zip › 3.jpg]

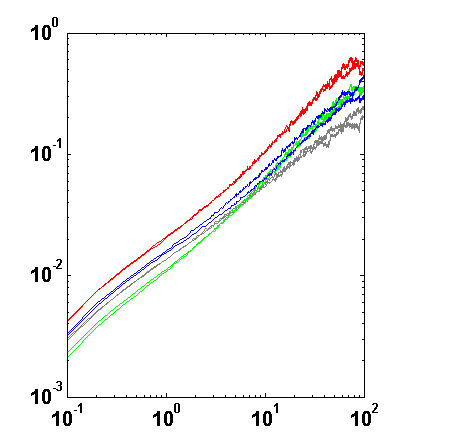

Supplement: Figure 2—figure supplement 1—source data 1. [file elife-78015-fig2-figsupp1-data1.zip › F2S1SD1.jpg]

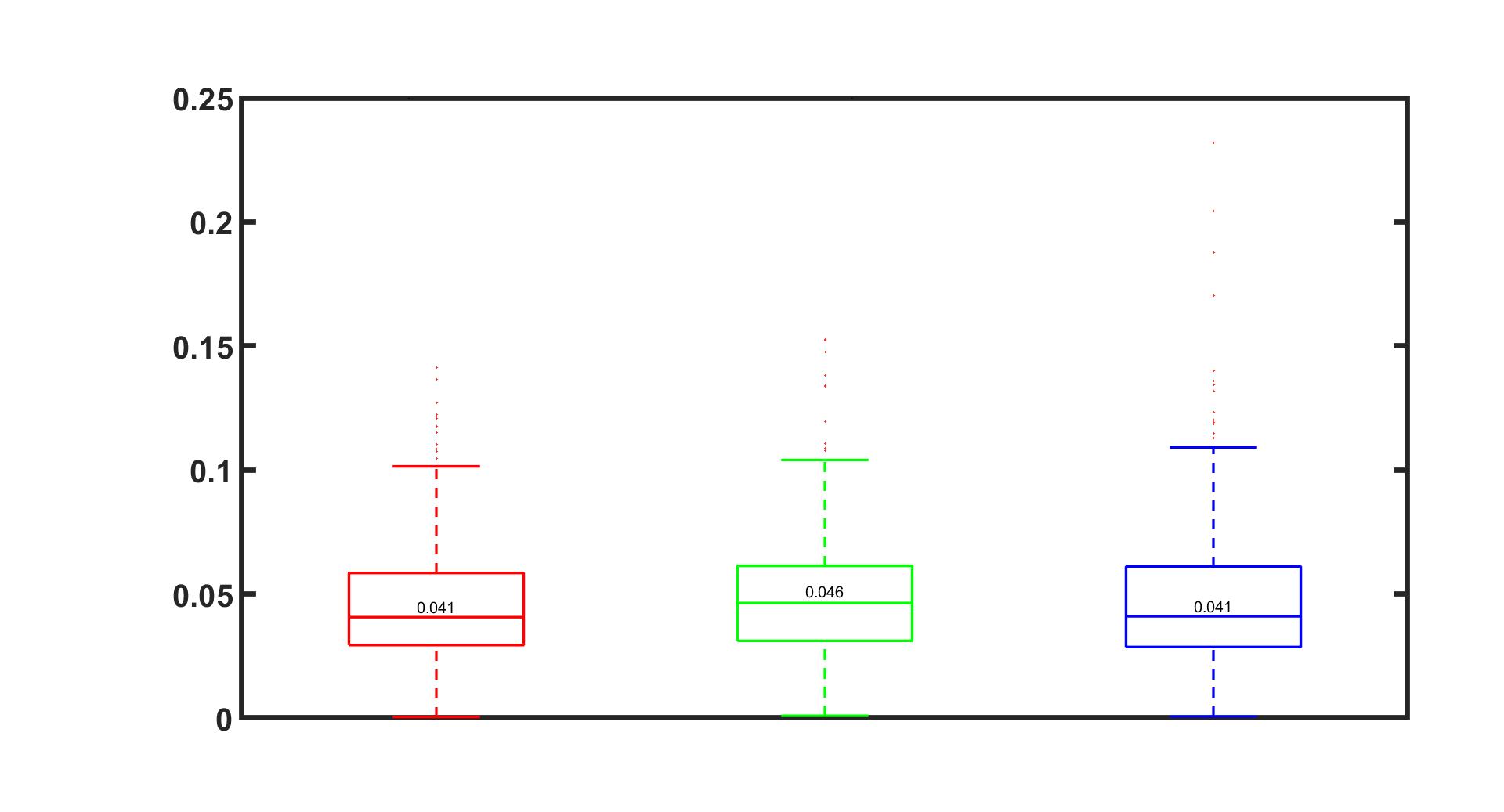

Supplement: Figure 2—figure supplement 1—source data 3. [file elife-78015-fig2-figsupp1-data3.zip › 1e27f3f9-9fe9-4ac0-88b0-a469c4768816.jpg]

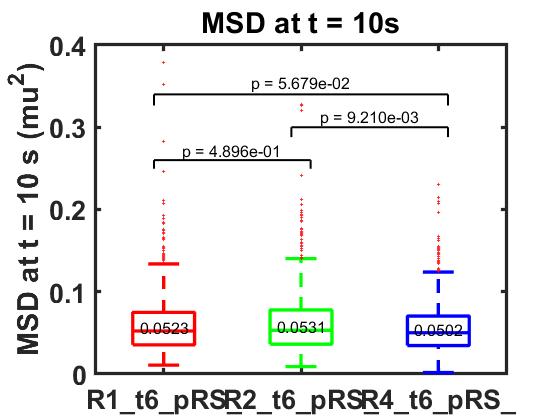

Supplement: Figure 2—figure supplement 1—source data 3. [file elife-78015-fig2-figsupp1-data3.zip › 27be947b-eb7b-47a3-a62f-9cf820359061.jpg]

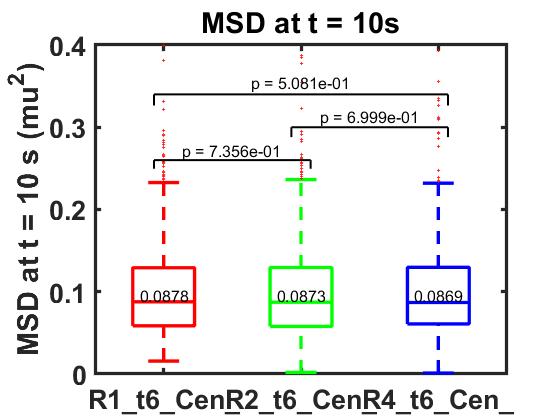

Supplement: Figure 2—figure supplement 1—source data 3. [file elife-78015-fig2-figsupp1-data3.zip › 8860edc2-4926-4f55-9ee4-c8ce7472b2b0.jpg]

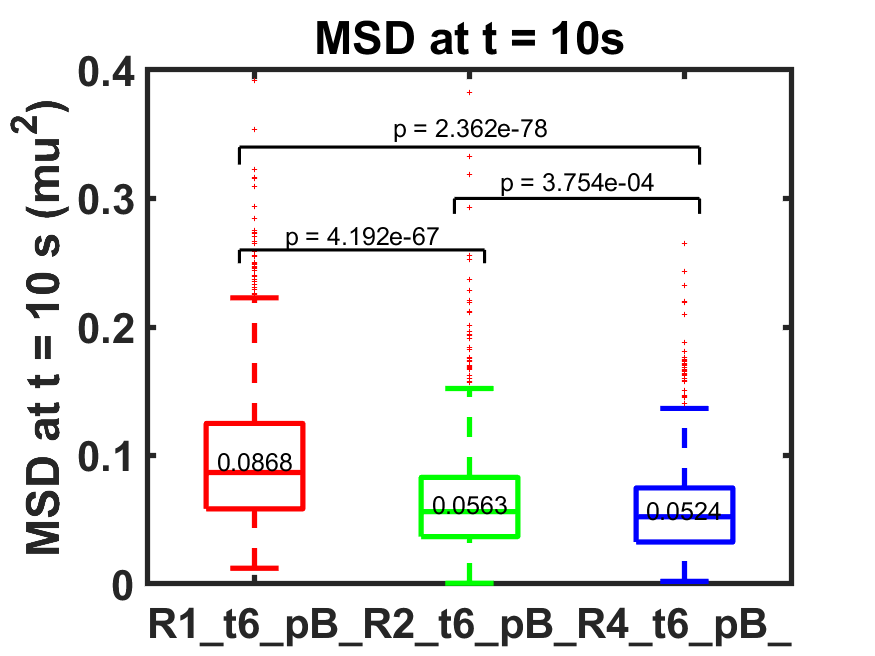

Supplement: Figure 2—figure supplement 1—source data 3. [file elife-78015-fig2-figsupp1-data3.zip › a560bf10-1411-48a5-aecf-be1ff5b7b37e.jpg]

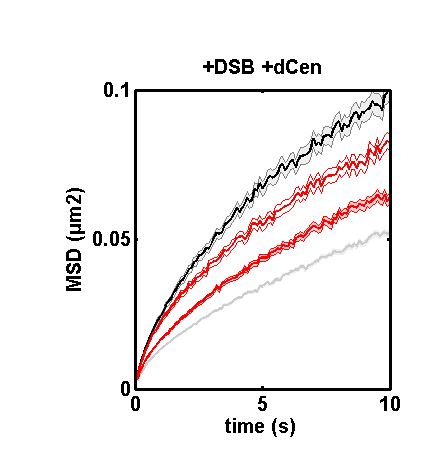

Supplement: Figure 3—source data 1. [file elife-78015-fig3-data1.zip › F3SD1.jpg]

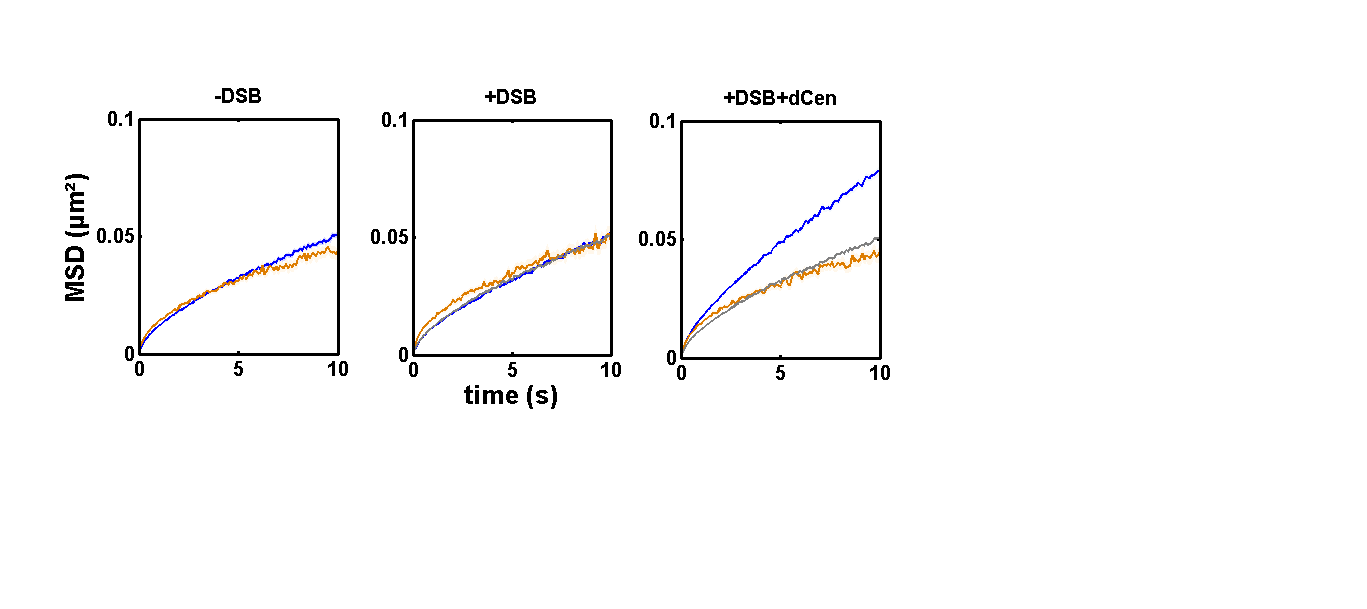

Supplement: Figure 3—source data 3. [file elife-78015-fig3-data3.zip › 2c07ce7e-7810-4f20-942a-52a0a1c45362.jpg]

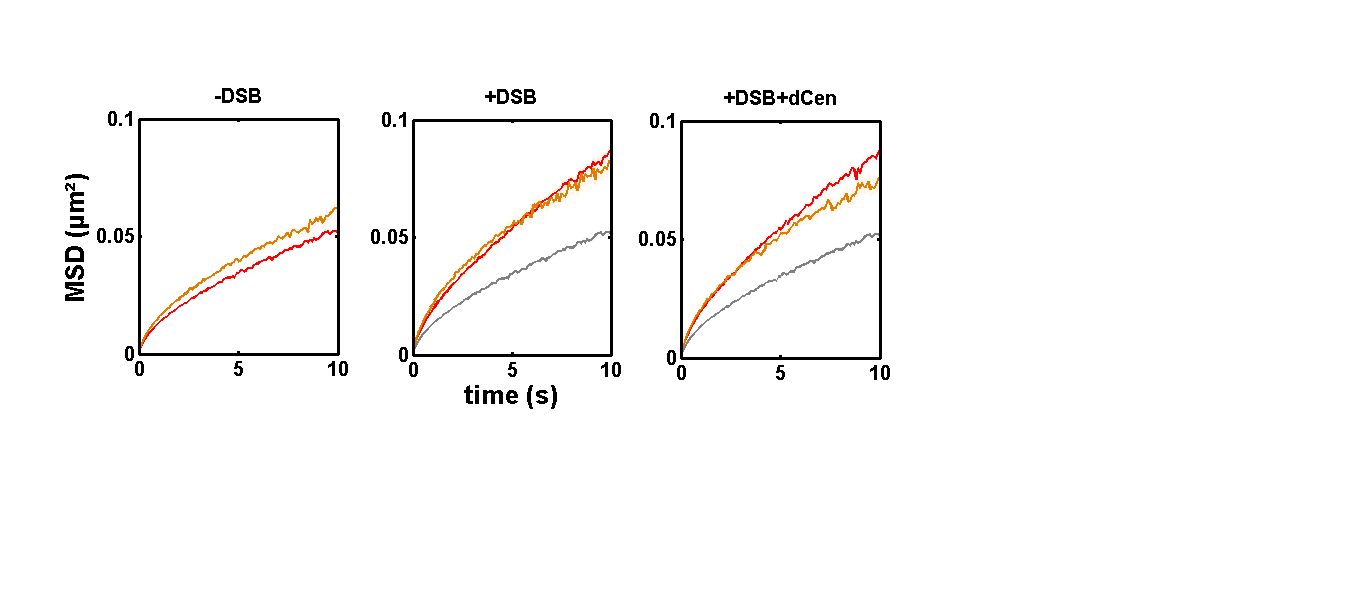

Supplement: Figure 3—source data 3. [file elife-78015-fig3-data3.zip › 9169068e-6f4b-412a-80d6-e7fb74b4a1c3.jpg]

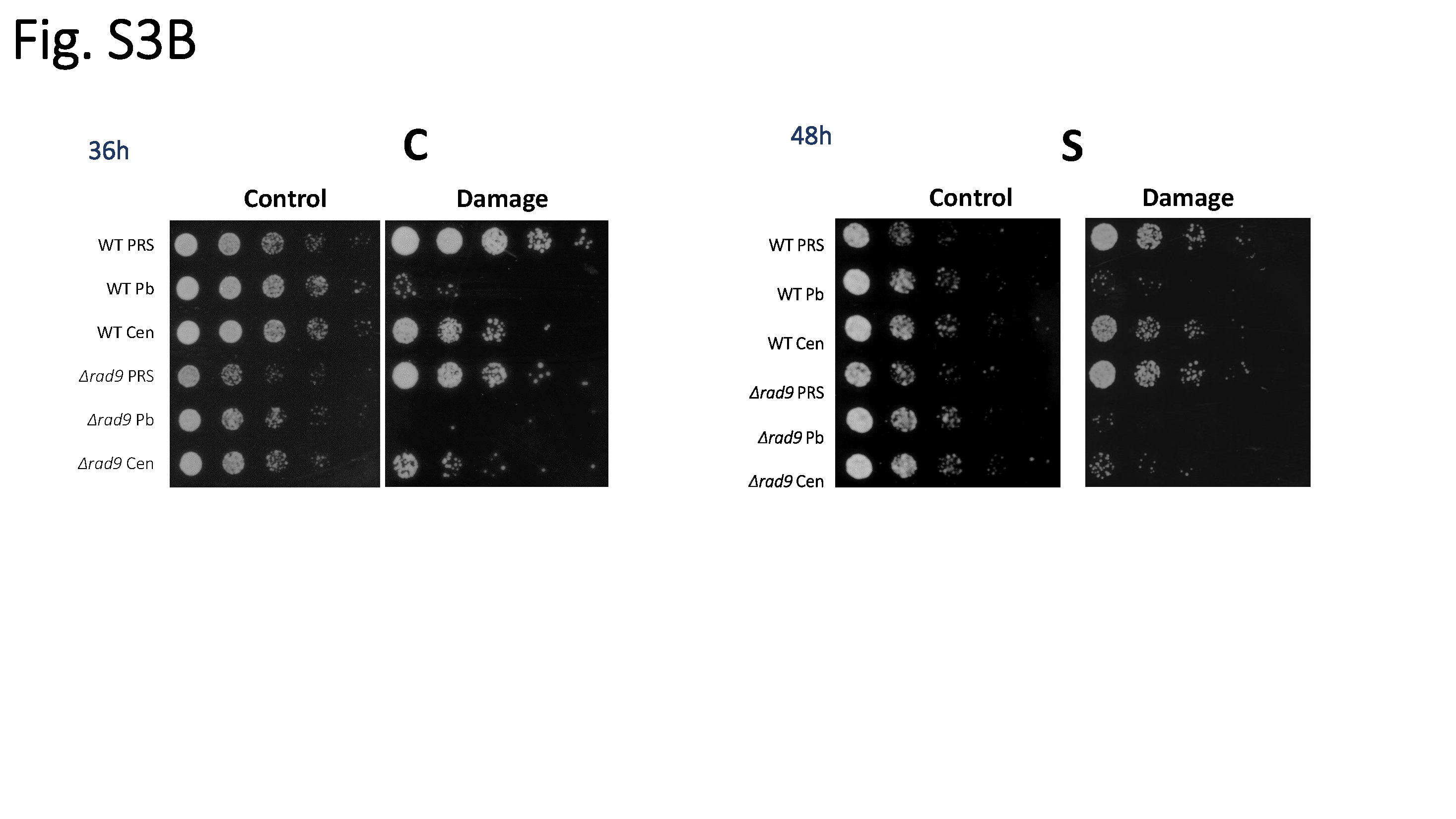

Supplement: Figure 3—figure supplement 1—source data 2. [file elife-78015-fig3-figsupp1-data2.zip › F3S1SD1.jpg]

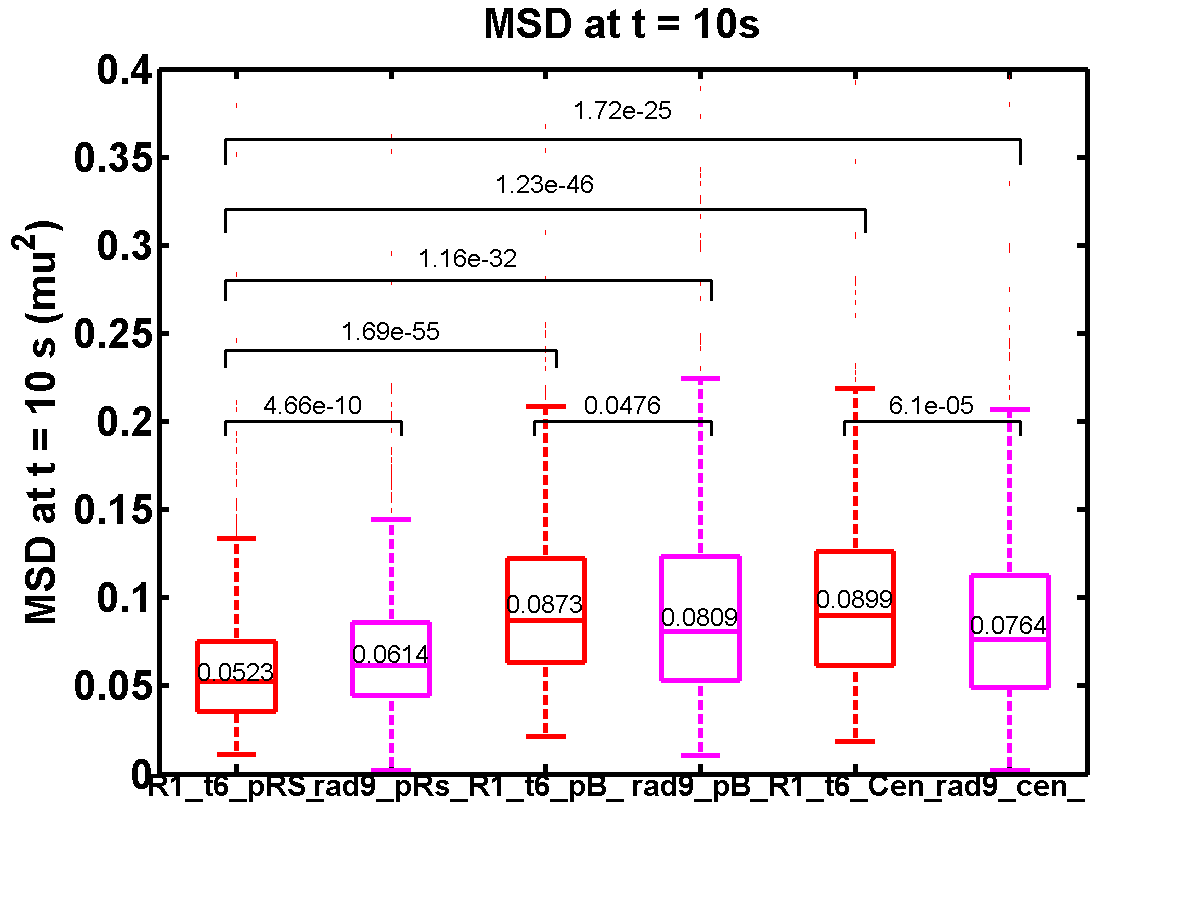

Supplement: Figure 3—figure supplement 1—source data 4. [file elife-78015-fig3-figsupp1-data4.zip › 60de0e39-7d85-45c7-95e4-f483c48fafb8.jpg]

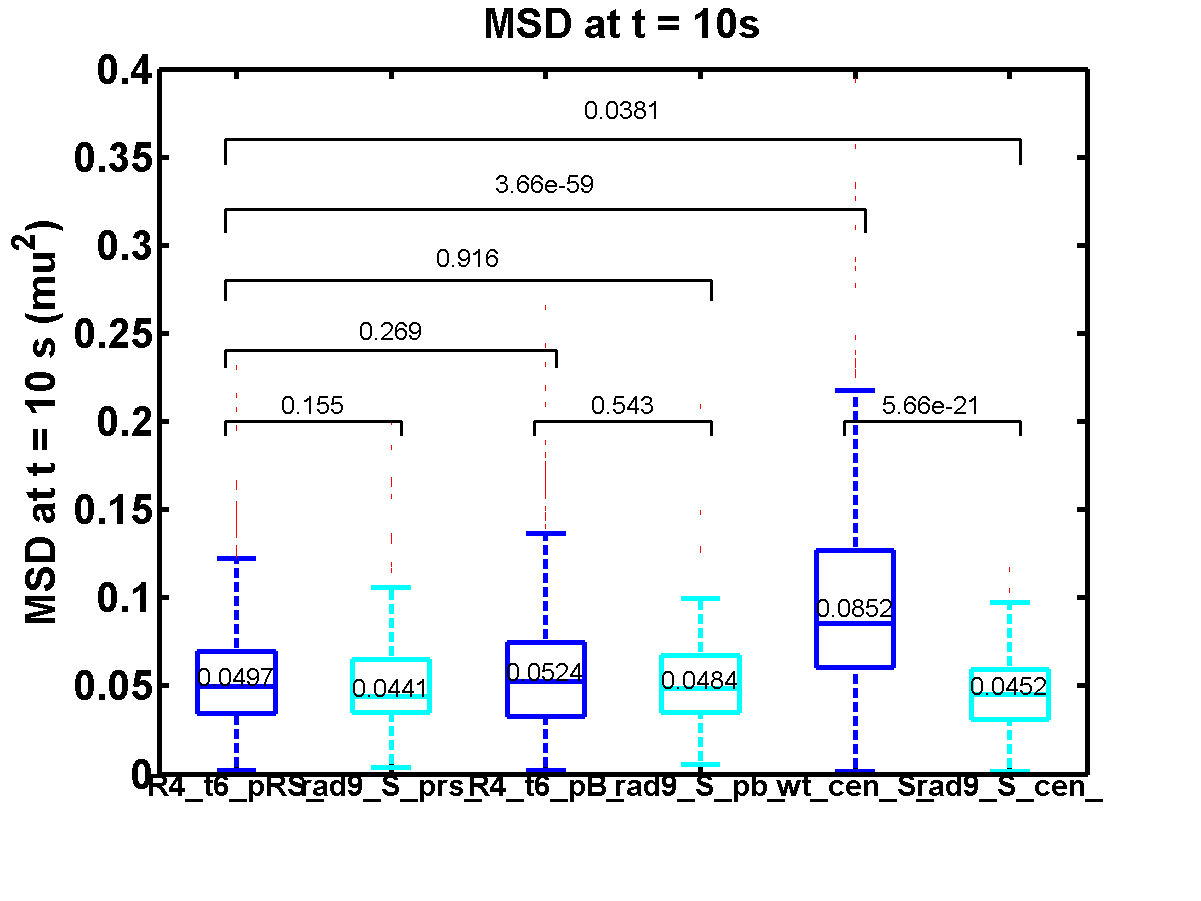

Supplement: Figure 3—figure supplement 1—source data 4. [file elife-78015-fig3-figsupp1-data4.zip › e9d20845-29f4-4abe-a964-d85900e418db.jpg]

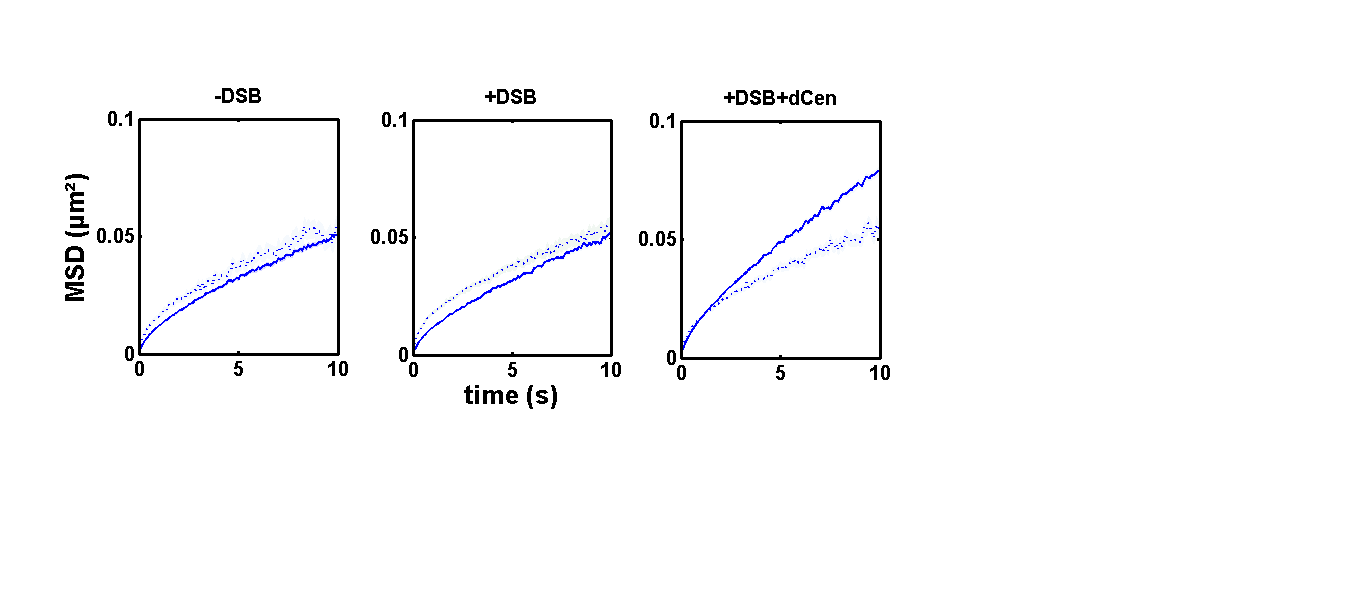

Supplement: Figure 4—source data 3. [file elife-78015-fig4-data3.zip › ec9f9c97-11de-4dfb-a29e-6621597dc373.jpg]

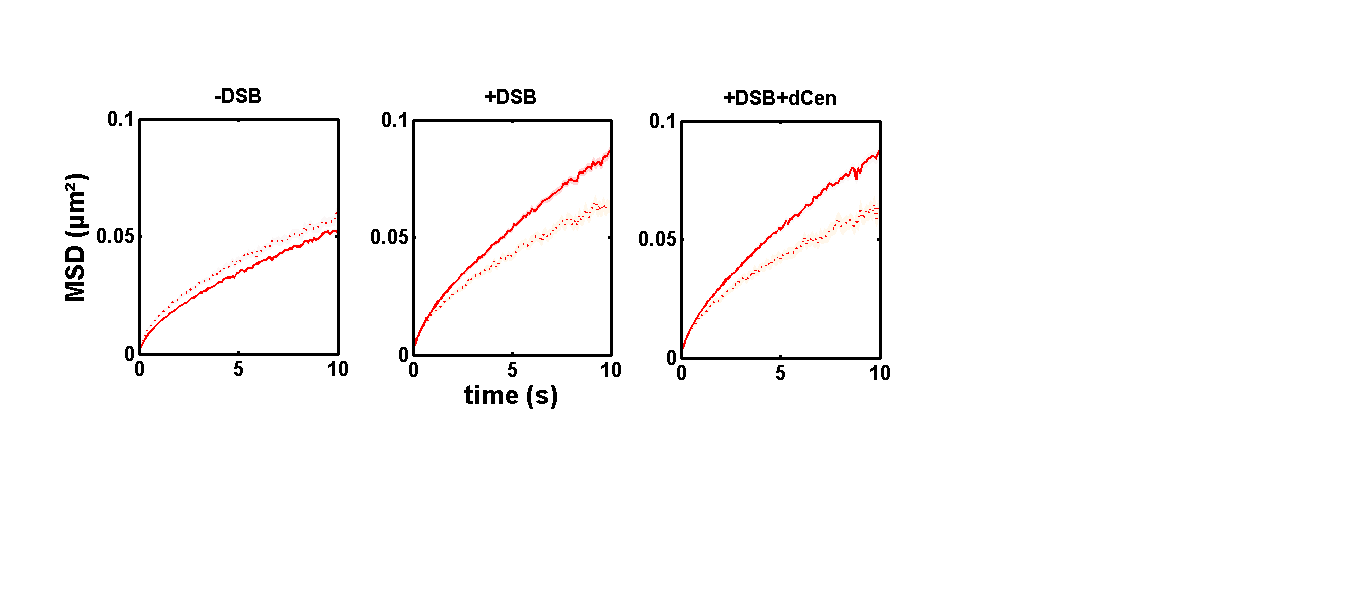

Supplement: Figure 4—source data 3. [file elife-78015-fig4-data3.zip › f9ea1a2c-ce3b-4f4b-9b3e-e071f5ae8a25.jpg]

# Fig. S4B

36h

**C**

**Control**

**Damage**

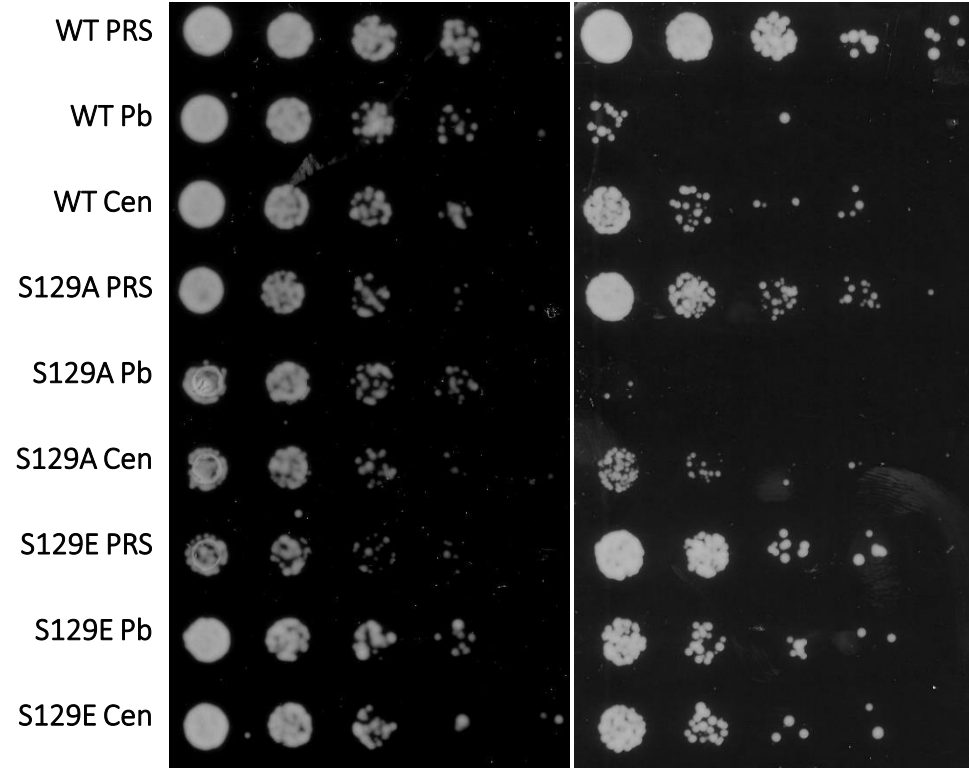

36h

**S**

**Control**

**Damage**

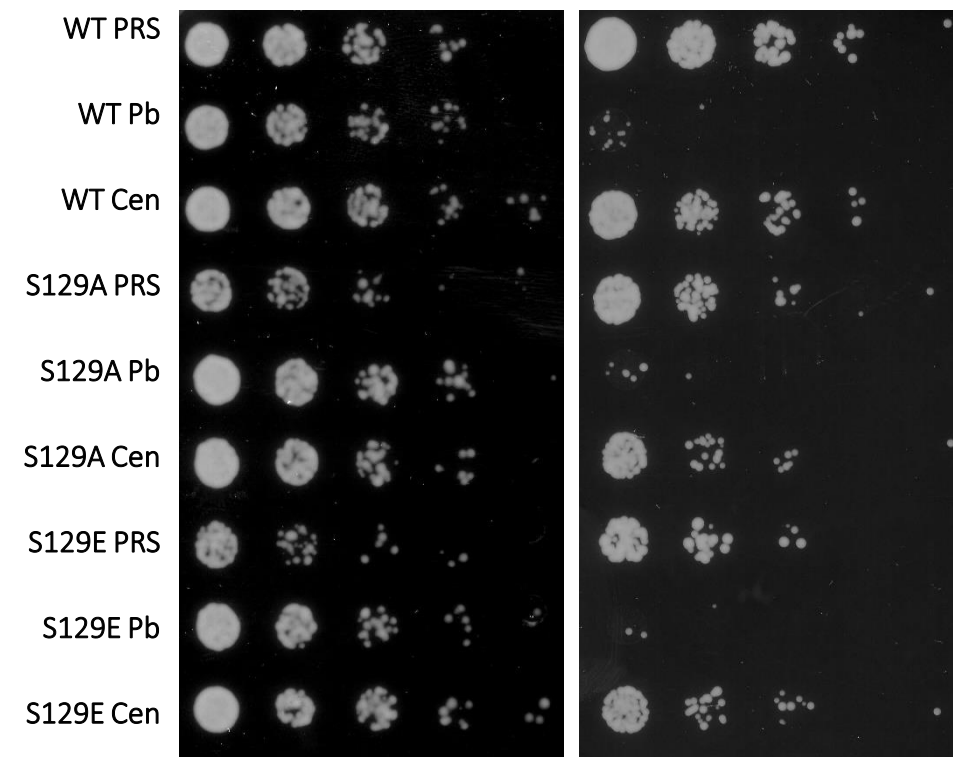

Supplement: Figure 4—figure supplement 1—source data 1. [file elife-78015-fig4-figsupp1-data1.pdf]

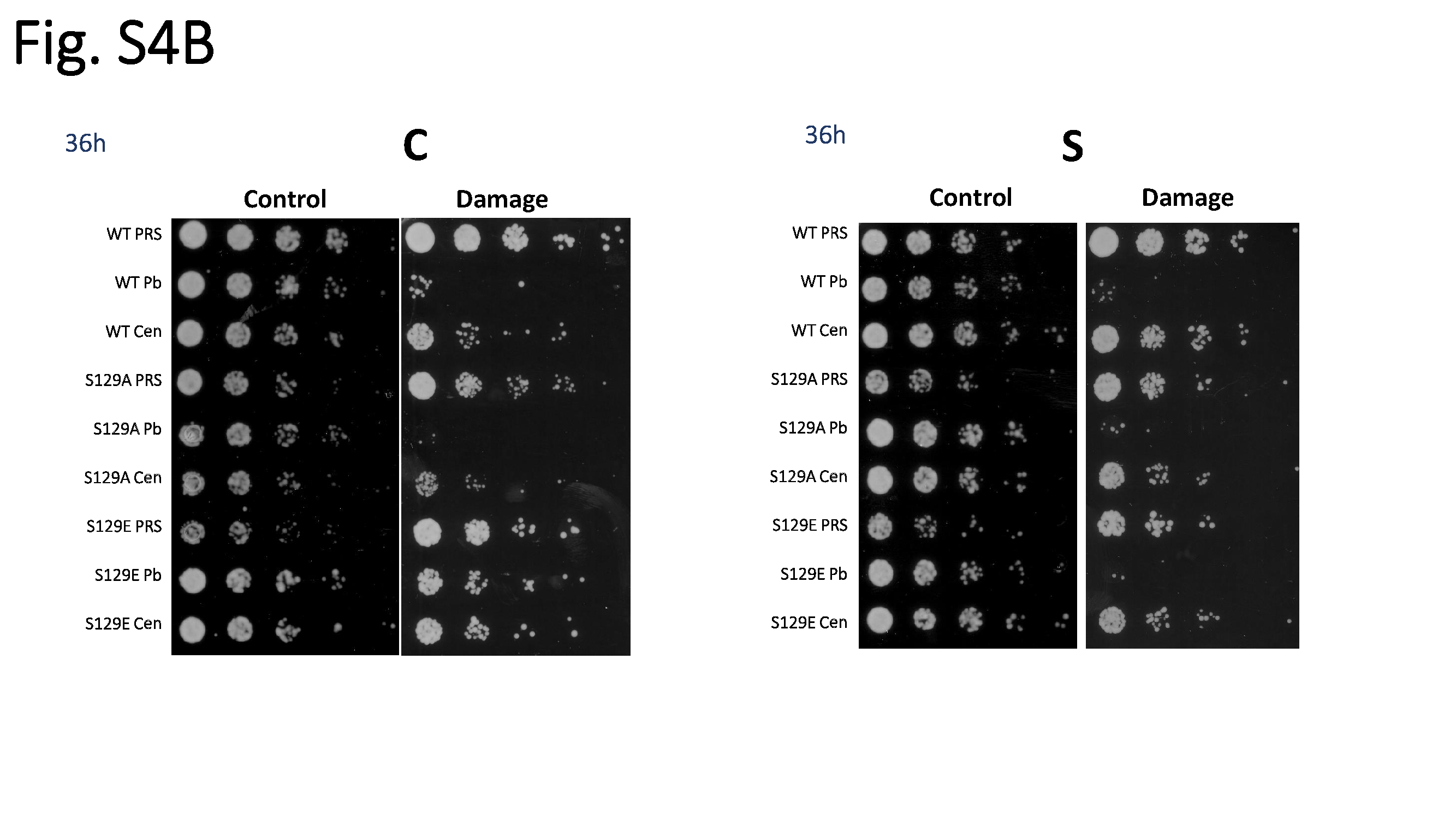

Supplement: Figure 4—figure supplement 1—source data 2. [file elife-78015-fig4-figsupp1-data2.zip › F4S1SD1.jpg]

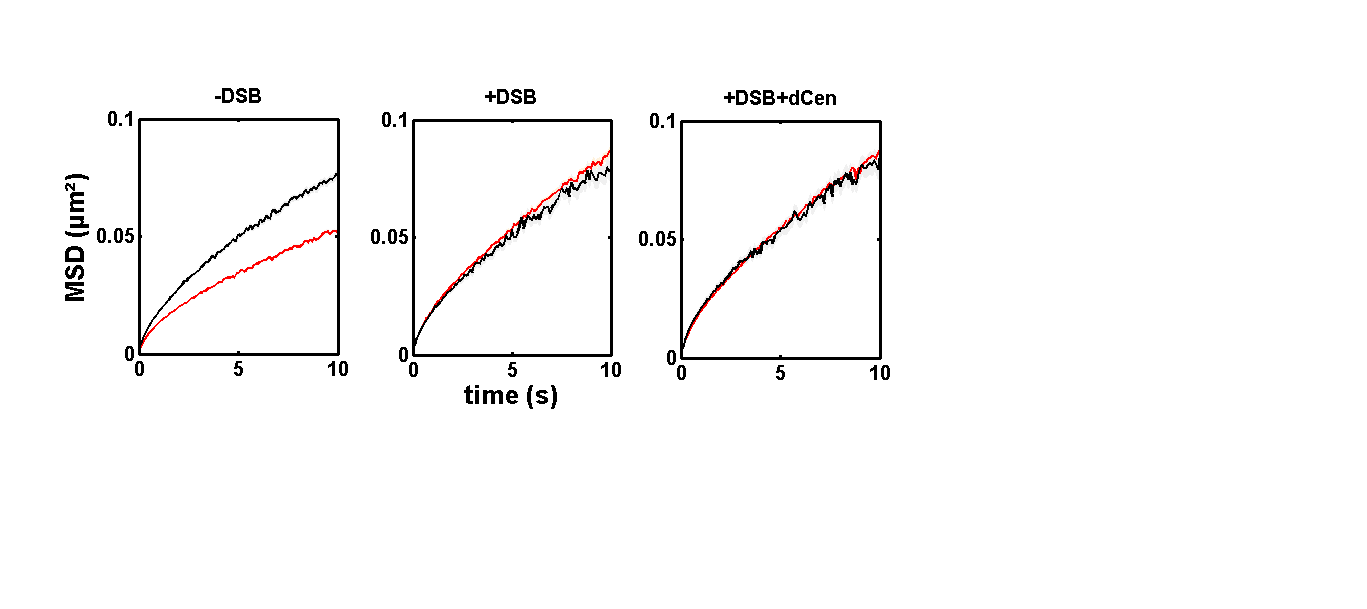

Supplement: Figure 4—figure supplement 1—source data 3. [file elife-78015-fig4-figsupp1-data3.zip › 1ed77dec-81dd-4eac-8ab3-4e9581a0fe15.jpg]

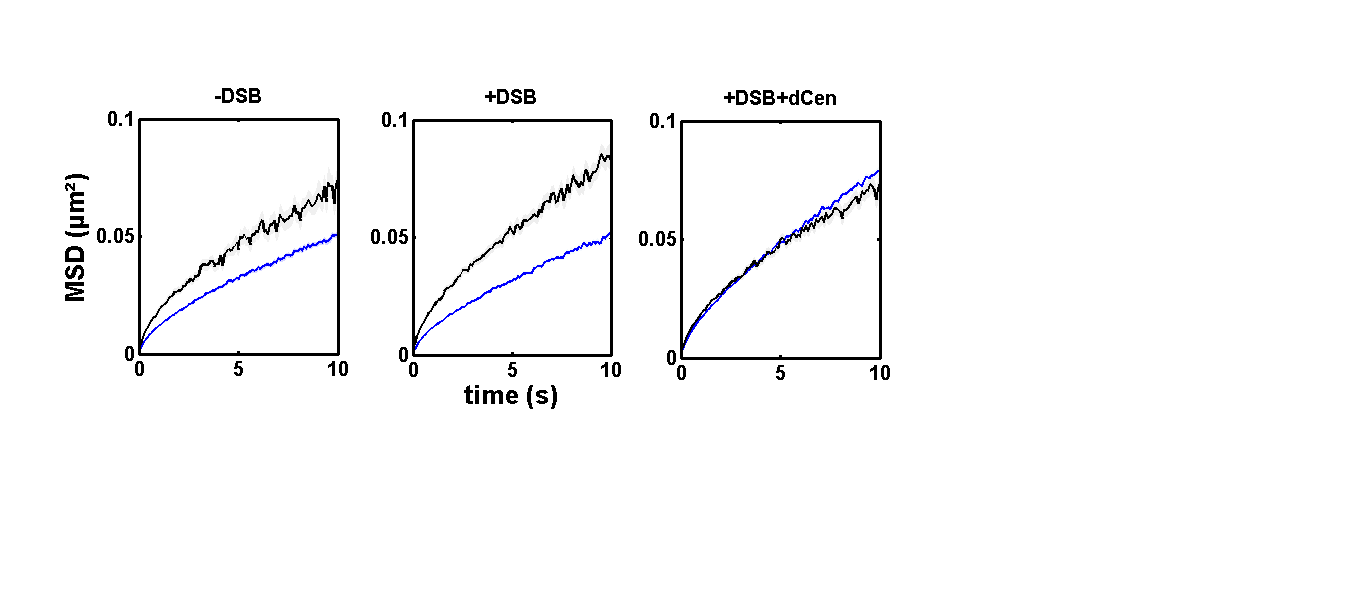

Supplement: Figure 4—figure supplement 1—source data 3. [file elife-78015-fig4-figsupp1-data3.zip › ffea2136-ebfc-4143-af54-765449e71fd7.jpg]

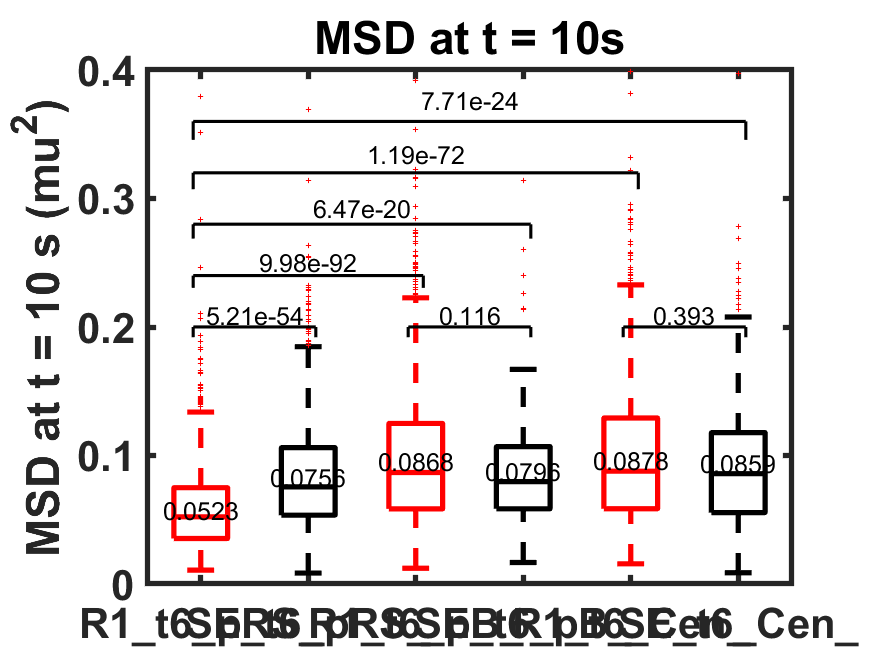

Supplement: Figure 4—figure supplement 2—source data 1. [file elife-78015-fig4-figsupp2-data1.zip › Figure 4- figure supplement 2/Figure 4- figure supplement 2 - source data 1.6.png]

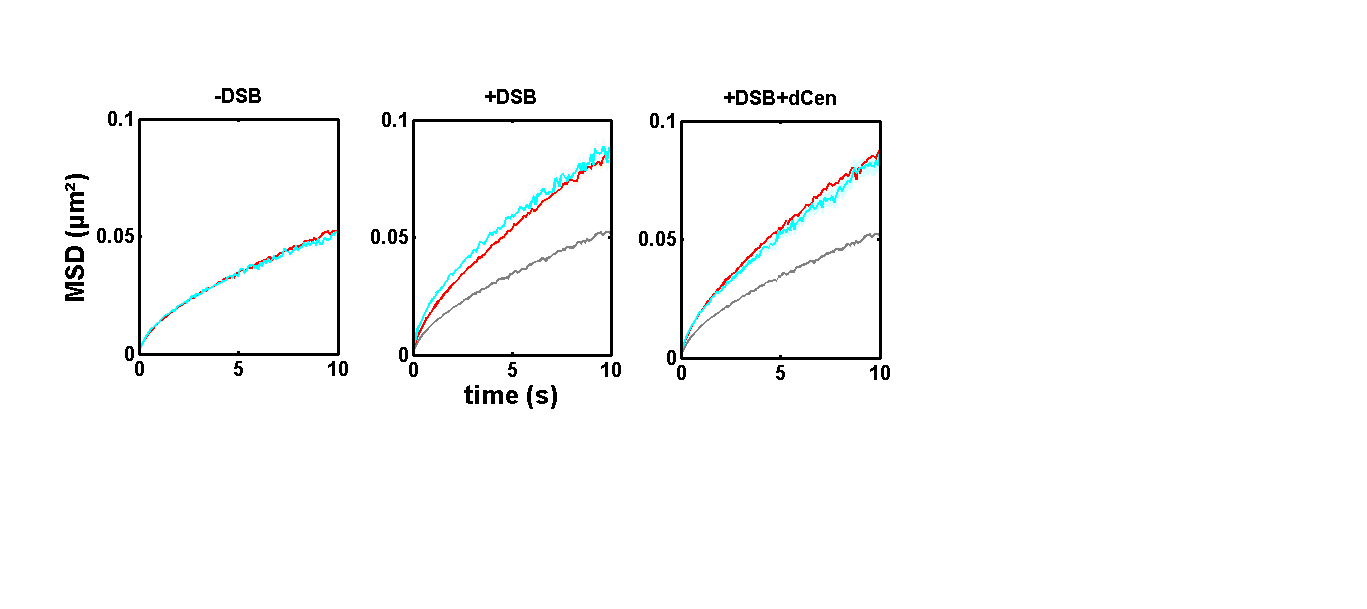

Supplement: Figure 5—source data 2. [file elife-78015-fig5-data2.zip › ac75e8f2-ab60-4155-a706-492659bbe94a.jpg]

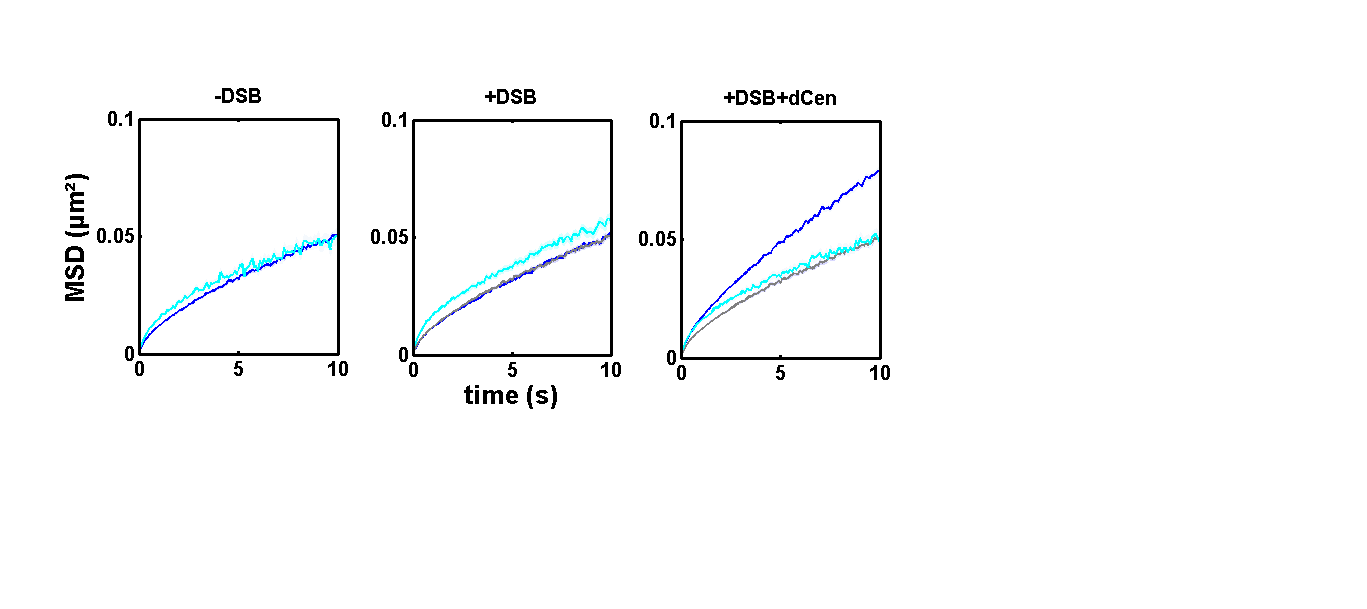

Supplement: Figure 5—source data 2. [file elife-78015-fig5-data2.zip › ebf6c43b-718f-48bc-b13b-7996acf3425e.jpg]

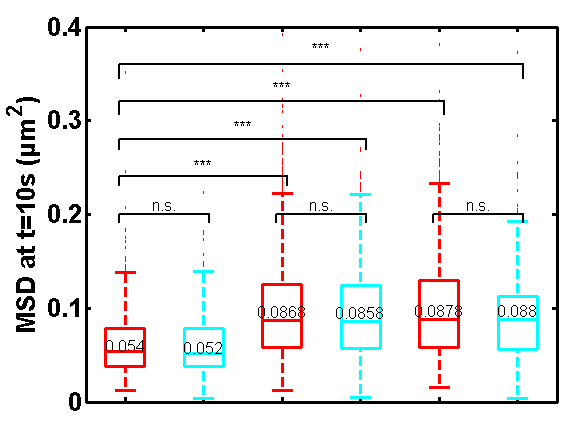

Supplement: Figure 5—source data 3. [file elife-78015-fig5-data3.zip › 07f4f397-26f1-42b3-a2d3-3fd29dd33b63.jpg]

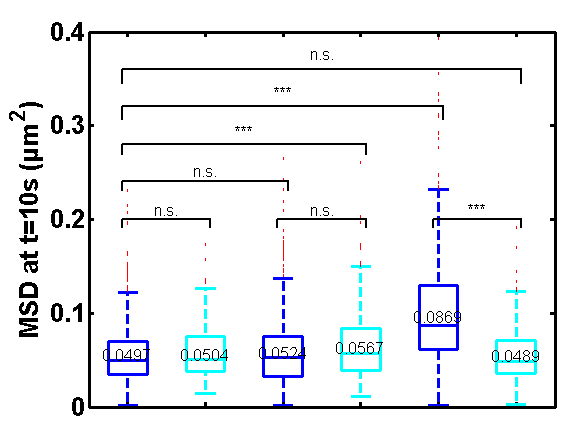

Supplement: Figure 5—source data 3. [file elife-78015-fig5-data3.zip › 41440aa0-5a82-4078-b3fc-6127642015f8.jpg]
