# Supplementary material for: Global chromatin mobility induced by a DSB is dictated by chromosomal conformation and defines the HR outcome
Source: eLife. 2022 Sep 20;11:e78015. doi: 10.7554/eLife.78015 (PMC9489209; doi:10.7554/eLife.78015)
Supplement: Figure 3—figure supplement 1—source data 1. [file elife-78015-fig3-figsupp1-data1.pdf]

Fig. S3B

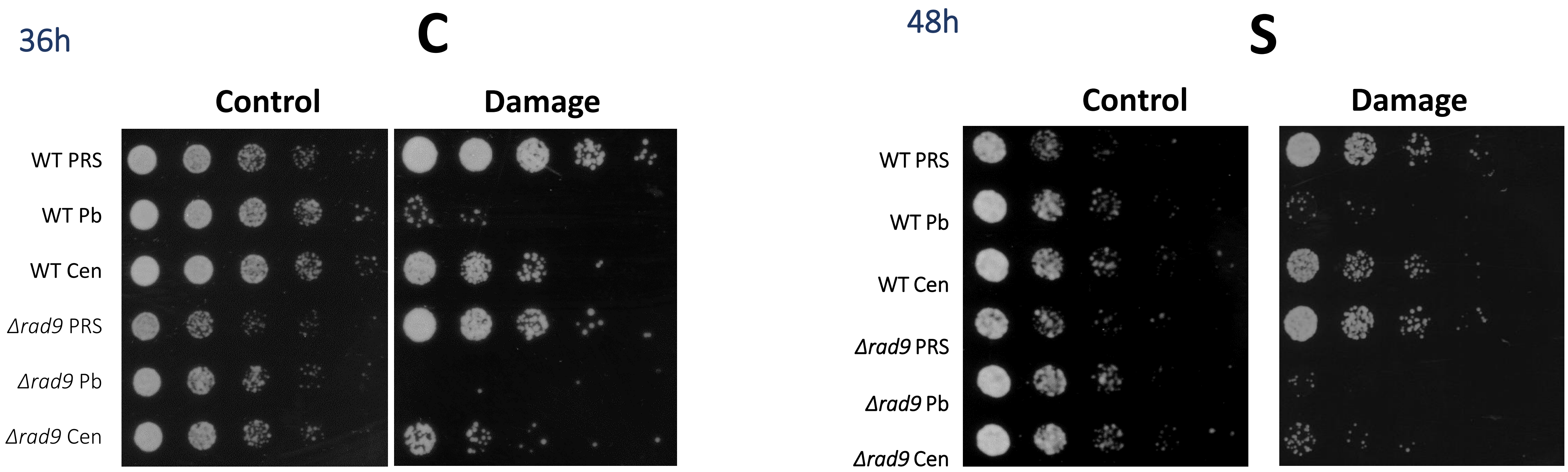
36h
C
Control
Damage
WT PRS
WT Pb
WT Cen
Δrad9 PRS
Δrad9 Pb
Δrad9 Cen
48h
S
Control
Damage
WT PRS
WT Pb
WT Cen
Δrad9 PRS
Δrad9 Pb
Δrad9 Cen
